# Cardiovascular Autonomic Modulation in Chronic Coronary Syndrome Following Percutaneous Coronary Intervention

**DOI:** 10.7759/cureus.65092

**Published:** 2024-07-22

**Authors:** Waqas Alauddin, Meenakshi Chaswal, Musharaf Bashir, Hermohander S Isser

**Affiliations:** 1 Physiology, Naraina Medical College and Research Centre, Kanpur, IND; 2 Physiology, Atal Bihari Vajpayee Institute of Medical Sciences and Dr. Ram Manohar Lohia Hospital, New Delhi, IND; 3 Physiology, Government Medical College, Srinagar, IND; 4 Cardiology, Vardhman Mahavir Medical College and Safdarjung Hospital, Delhi, IND

**Keywords:** parasympathetic reactivity, sympathetic reactivity, autonomic dysfunction, chronic coronary syndrome (ccs), percutaneous coronary intervention (pci), cardiovascular reflex tests, sudden cardiac death

## Abstract

Introduction

The risk of sudden death in patients with chronic coronary syndrome (CCS) is increased by unbalanced cardiovascular autonomic function. Since myocardial ischemia appears to be the cause of this condition of autonomic dysregulation, treating this condition should improve and correct the autonomic functions. Improving myocardial perfusion by PCI might have beneficial effects on the recovery of autonomic balance in ischemia-triggered autonomic dysregulation.

Objective

In the present study, autonomic modulation in patients with CCS was evaluated before and after percutaneous coronary intervention (PCI) using cardiovascular reflex tests.

Methods

A total of 30 CCS patients were recruited from the cardiology outpatient department. The patients were tested with cardiovascular reflex tests (lying to standing, 30:15 ratio, Valsalva ratio, isometric handgrip test, and deep breathing test) before and after PCI. The licensed statistical software SPSS version 21.0 was used to compile and analyse the data.

Results

Out of 30 patients, parasympathetic reactivity tests conducted post-PCI were significantly higher as compared to pre-PCI patients: (1) lying to standing - 30:15 ratio (1.17± 0.102 versus 1.03± 0.064, p=0.000); (2) Valsalva ratio (1.42±0.276 versus 1.02±0.133, p=0.000), (3) delta heart rate in deep breathing test (17.23± 3.004 bpm versus 7.85± 4.076 bpm, p=0.000), and (4) expiration to inspiration (E:I) ratio (1.25± 0.050 versus 1.11± 0.064, p=0.000. Among sympathetic reactivity tests, lying to standing test for fall in systolic blood pressure was significantly higher in the pre-PCI state than post-PCI (-20.73± 10.29 versus -2.33± 7.67, p=0.000). The rise in DBP of the isometric handgrip test was significantly higher in post-PCI compared to pre-PCI patients (36.73±8.39 mm Hg versus 16.63±8.47 mm Hg, p=0.000).

Conclusion

Resting autonomic tone as determined by cardiovascular reflex testing reveals an increase in both parasympathetic and sympathetic reactivity following PCI in CCS, according to the findings of this preliminary study. As a result, we propose that noninvasive procedures like cardiovascular reflex tests be used to stratify the likelihood of illness development in the future.

## Introduction

Coronary artery disease (CAD) is the world's leading cause of illness and death [1]. It is an epidemic in India, accounting for over 23% of overall fatalities and nearly 32% of deaths in the adult population, with an increase in the last 10 years. According to the World Health Organization (WHO) and the Global Burden of Disease Study, coronary artery disease is a major disease burden in India, resulting in an increase in both loss of years of life and disability-adjusted years of life [2]. The most common type of stable coronary artery disease is chronic coronary syndrome (CCS) [3]. The risk of adverse cardiovascular outcomes, such as myocardial infarction and cardiac mortality, increases significantly when CCS is present [4]. CCS often manifests as uneasiness, discomfort, or squeezing pain in the chest that arises or is increased by effort and is relieved by rest or nitroglycerin [5]. Anginal pain, which is characteristic of CCS, is caused by myocardial ischemia exacerbated by an imbalance in the consumption and supply of oxygen for the myocardium as a result of excessive atherosclerotic constriction of coronary arteries. This results in autonomic dysfunction as a result of this chronic state of ischemia and hypoperfusion [6-8]. Previously, a few studies found a decrease in HRV index in these individuals [9]; others found that patients with CCS often had greater sympathetic influence, and it appears that autonomic dysfunction is caused by the chronic state of ischemia myocardium [10]. Because myocardial ischemia seems to be the origin of this autonomic dysregulation, removing this cause should help to correct and improve autonomic functions. When compared to pharmacologic therapy, revascularization procedures have been linked to a higher improvement in angina symptoms. As a result, increasing myocardial perfusion with percutaneous coronary revascularization (PCI) may help with autonomic rebalancing in patients with ischemia-induced autonomic dysregulation.

There are no studies that we are aware of that have looked at the cardiovascular reflex test in patients with CCS and the impact of PCI therapy on it. Apart from that, most previous investigations in CCS patients solely evaluated heart rate variability to measure cardiac autonomic function. The current study was conducted to investigate the autonomic regulation of the cardiovascular system in patients with CCS and the impact of PCI revascularization on it in order to resolve these gaps.

## Materials and methods

This was a pre- and post-intervention study design. After receiving ethical approval from the Institutional Ethical Committee with approval number IEC/VMMC/SJH/Thesis/October/2017-177 and patients' written consent, the study was carried out in the Autonomic Function Test laboratory of the Department of Physiology, Vardhman Mahavir Medical College and Safdarjung Hospital, New Delhi. The study included 30 patients with CCS recruited from the Cardiology outpatient department of the same institute comprising males and females ranging in age from 45 to 70 years. Patients with a history of autoimmune disorders or collagen disease, uncontrolled hypertension and diabetes, left ventricular dysfunction with ejection fraction (EF) 35%, heart failure, congenital heart disease, valvular heart disease, or arrhythmias, abnormal thyroid function test, liver function test, and renal function tests, symptomatic autonomic neuropathy or neuropsychiatric disorders, or individuals with a history of substance abuse were excluded from the study.

To eliminate reaction differences owing to circadian variations, all tests were conducted in thermo-neutral circumstances and at the same time of day on all volunteers, i.e. in the morning hours. Prior to the test, the volunteers were told to avoid alcoholic beverages and smoking as well as stimulants such as tea, and coffee and to eat a light breakfast in the morning. BIOPAC MP 150 and the Student's Physiograph were utilised for these examinations.

The sympathetic and parasympathetic responses were assessed using a standard battery of cardiovascular reflex tests [11].

Sympathetic reactivity was assessed by systolic blood pressure response during lying to standing test, and diastolic blood pressure response during isometric handgrip test. Parasympathetic reactivity was assessed by the E:I ratio (expiration to inspiration) during the deep breathing test, the Valsalva ratio during the Valsalva manoeuvre and the 30:15 ratio during the lying to standing test.

Test protocols

Lying to Standing Test

The subject's supine blood pressure was taken, and then he was asked to get up in three seconds. Within five minutes of standing, the highest drop in systolic blood pressure was seen.

The 30:15 ratio was determined using the maximum RR interval of 30 seconds and the minimum RR interval of 15 seconds. A systolic blood pressure drop of less than 10 mmHg and a 30:15 ratio of more than 1.04 are considered normal.

Deep Breathing Test

For 30 seconds, an ECG baseline was collected. The patient was told to breathe gently and deeply at a rate of six cycles per minute. The longest RR interval during expiration and the smallest RR interval during inspiration were used to compute the E:I ratio. Each subject's average value of six cycles was calculated. The average E:I ratio is more than 1.21.

Valsalva Manoeuvre

The baseline ECG was collected for this procedure. The participant was then instructed to blow into a mouthpiece coupled to a sphygmomanometer for 15 seconds, raising the pressure to 40 mmHg. The Valsalva ratio was determined using the highest RR interval in Phase IV and the shortest RR interval in Phase II. The average VR ratio is more than 1.21.

Handgrip Test

The baseline blood pressure was taken first. The individual was then told to hold the hand grip dynamometer for four minutes at 30% of their maximum voluntary contraction (MVC). During the test, the rise in diastolic pressure was measured. A rise in diastolic blood pressure of more than 10 mmHg is considered typical.

Data analysis

MS Excel was used to assemble and enter data, and licenced statistical software SPSS version 21.0 (IBM Corp., Armonk, NY) was used to analyse it. Mean and standard deviation (SD) were used to present the data. The paired Student t-test or non-parametric test was used to determine the statistical significance of the changes in the pre- and post-intervention tests. A p-value of less than or equal to 0.05 was used as the level of significance.

## Results

The present study comprised 30 patients with CCS (both sexes) who were receiving percutaneous coronary intervention between the ages of 45 and 70. In terms of the subjects' basic characteristics, as depicted in Table 1, patients with CCS ranged in age from 53.7 ± 8.9 years. In terms of gender distribution, there were 22 (73.3%) males and eight (26.67%) females. The average BMI of the study's participants was 24.23±2.59 kg/m2. In terms of co-morbidities, 3 (10%) of CCS patients were diabetics, 5 (16.66%) were hypertensives, and 14 (46.66%) had both diabetes and hypertension. There were 17 patients (56.66%) who had a smoking history and four (13.33%) with a history of both alcohol and smoking.

**Table 1:** Basal characteristics of chronic coronary syndrome patients Values are expressed as mean ± SD or number (%). BMI: body mass index; n: number

Parameters	Values
Age (yrs)	53.7 ± 8.9
Male, n (%)	22 (73.3%)
Female, n (%)	8 (26.67%)
BMI (kg/m^2^)	24.23 ± 2.59
History of diabetes, n (%)	3 (10%)
History of hypertension, n (%)	5 (16.66%)
History of hypertension and diabetes, n (%)	14 (46.66%)
Smokers, n (%)	17 (56.66%)
Smokers + alcoholics, n (%)	4 (13.33%)

**Figure 1:**
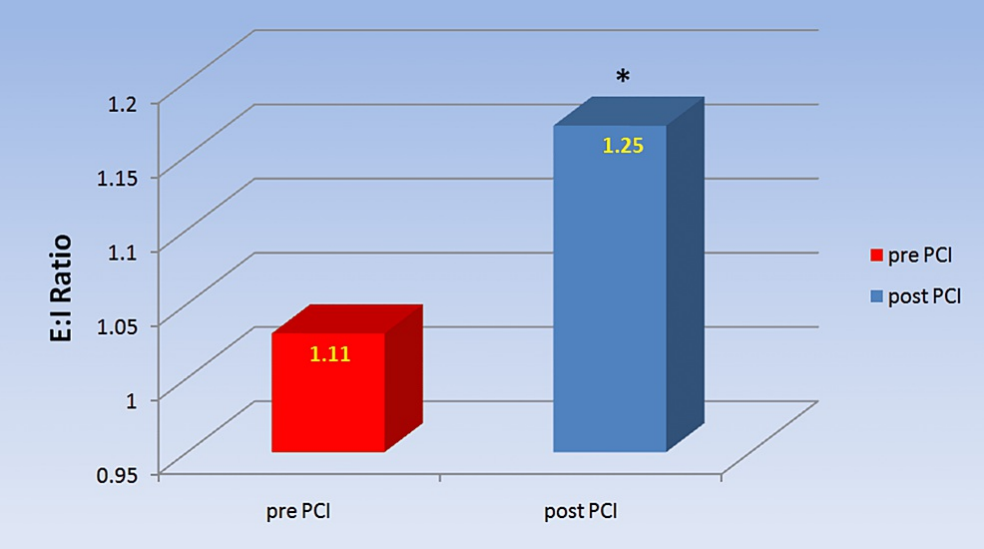
Comparison of E:I ratio in chronic coronary syndrome patients pre- and post-PCI Post-PCI showed significant improvement in E:I ratio as compared to pre-PCI. *a p-value<0.05 was considered statistically significant. E:I ratio: expiration/inspiration ratio; PCI: percutaneous coronary intervention

Regarding the parasympathetic reactivity test, the E:I ratio in post-PCI patients was significantly higher than in pre-PCI patients (1.25±0.050 versus 1.11±0.064, p=0.000), as seen in Figure 1 and Table 2.

**Table 2:** Comparison of E:I ratio and delta heart rate of deep breathing test of chronic coronary syndrome patients pre- and post-PCI The values are expressed as mean ± SD. *a p-value<0.05 was considered statistically significant. ∆ HR: delta heart rate; E:I ratio: expiration/inspiration ratio; bpm: beats per minute; n: number; PCI: percutaneous coronary intervention

Parameter	Pre-PCI (n=30)	Post-PCI (n=30)	p-value
E:I ratio	1.11± 0.064	1.25± 0.050	0.000*
∆ HR (bpm)	7.85± 4.076	17.23± 3.004	0.000*

**Figure 2:**
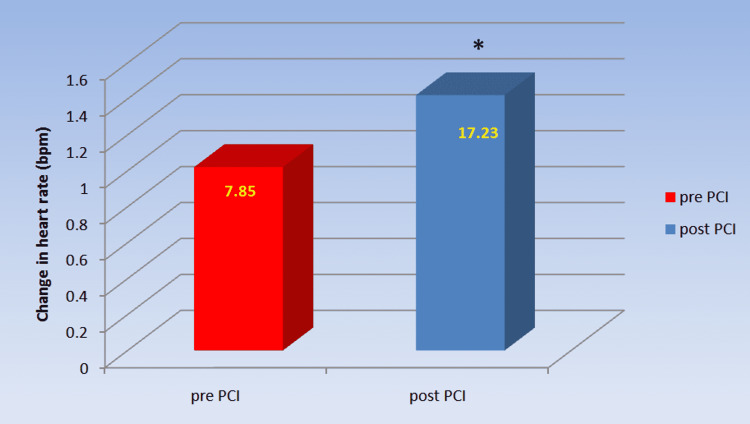
Comparison of change in delta heart rate in chronic coronary syndrome patients pre- and post-PCI Post-PCI showed significant improvement in delta heart rate as compared to pre-PCI. *a p-value<0.05 was considered statistically significant. PCI: percutaneous coronary intervention

The delta heart rate of post-PCI patients was also significantly greater than that of pre-PCI patients (17.23±3.004 against 7.85±4.076 bpm, p=0.000) as shown in Table 2 and Figure 2. 

Table 3 and Figure 4 show that the 30:15 ratio, which also shows parasympathetic reactivity, was significantly higher in post-PCI patients than in pre-PCI patients (1.17±0.102 versus 1.03±0.064, p=0.000)

**Table 3:** Comparison of 30:15 ratio (lying to standing test) of chronic coronary syndrome patients pre- and post-PCI Values are expressed as mean ± SD *a p-value<0.05 was considered statistically significant. n: number; PCI: percutaneous coronary intervention

Parameter	pre-PCI (n=30)	post-PCI (n=30)	p-value
30:15 ratio	1.03± 0.064	1.17± 0.102	0.000*

**Figure 3:**
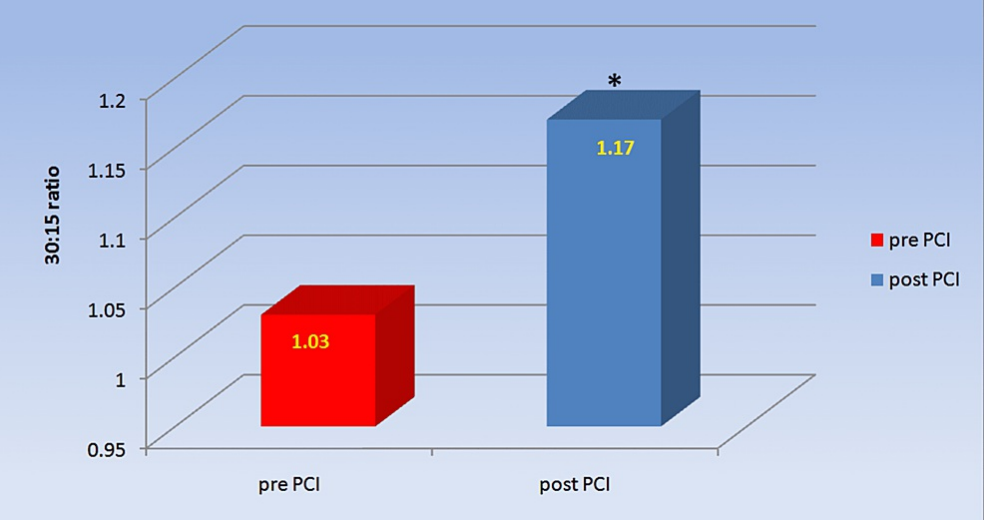
Comparison of 30:15 ratio in chronic coronary syndrome patients before and after PCI Post-PCI showed significant improvement in 30:15 ratio as compared to pre-PCI. *p-value<0.05 was considered statistically significant. PCI: percutaneous coronary intervention

**Figure 4:**
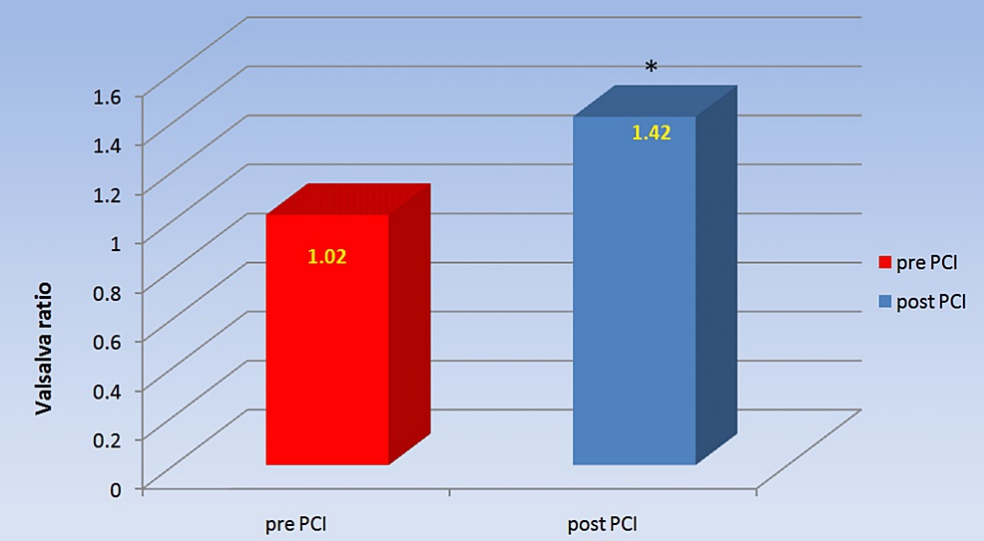
Comparison of Valsalva ratio in chronic coronary syndrome patients pre- and post-PCI Post-PCI patients showed significant improvement in Valsalva ratio as compared to pre-PCI patients. *a p-value<0.05 was statistically significant. PCI: percutaneous coronary intervention

As shown in Table 4 and Figure 4, the Valsalva ratio, which also represents parasympathetic reactivity, was significantly higher in post-PCI patients than in pre-PCI patients (1.42±0.276 versus 1.02±0.133, p=0.000).

**Table 4:** Comparison of Valsalva ratio of chronic coronary syndrome patients pre- and post-PCI Values are expressed as mean ± SD. *a p-value<0.05 was considered statistically significant. n: number; PCI: percutaneous coronary intervention

Parameter	pre-PCI (n=30)	post-PCI (n=30)	p-value
Valsalva ratio	1.02±0.133	1.42±0.276	0.000*

**Figure 5:**
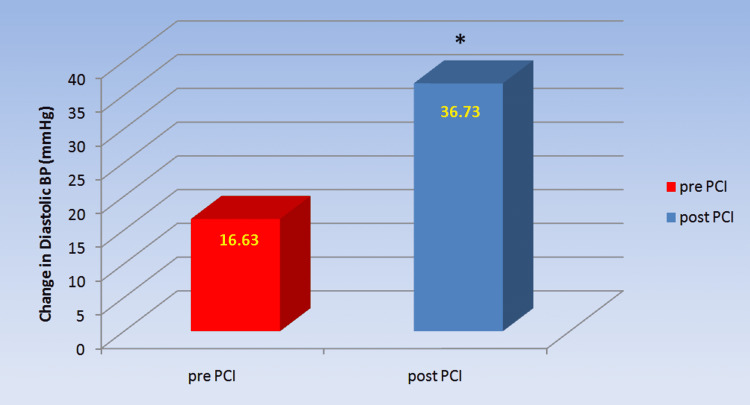
Rise in diastolic blood pressure measured by isometric handgrip test in chronic coronary syndrome patients pre- and post-PCI Post-PCI showed significant improvement in diastolic blood pressure as compared to pre-PCI. *a p-value<0.05 was considered statistically significant. PCI: percutaneous coronary intervention

Regarding the isometric hand grip test, the change in diastolic blood pressure illustrates sympathetic reactivity tests, as shown in Table 5 and Figure 5. The rise in DBP of IHT was considerably larger in post-PCI patients compared to pre-PCI patients (36.73±8.39 mm Hg versus 16.63±8.47 mmHg, p=0.000).

**Table 5:** Comparison of change in diastolic blood pressure in isometric handgrip test of chronic coronary syndrome patients pre- and post-PCI The values are expressed as mean ± SD. *a p-value<0.05 was considered statistically significant. ∆ DBP: delta diastolic blood pressure; PCI: percutaneous coronary intervention

Parameter	pre-PCI (n=30)	post-PCI (n=30)	p-value
∆ DBP (mmHg)	16.63±8.47	36.73±8.39	0.000*

**Figure 6:**
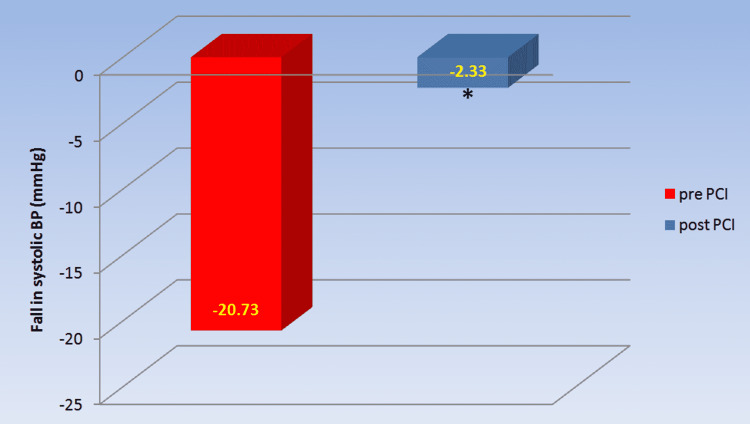
Comparison of change in ∆ SBP with time during lying to standing test in chronic coronary syndrome patients pre-and post-PCI Post-PCI showed significant improvement in change in ∆ SBP compared to pre-PCI. *a p-value<0.05 was considered statistically significant. ∆ SBP: delta systolic blood pressure; PCI: percutaneous coronary intervention

In the lying to standing test, the decrease in systolic blood pressure depicts sympathetic reactivity tests as shown in Table 6 and Figure 6. The fall in SBP during LST in pre-PCI patients was substantially higher than in post-PCI patients (-20.73±10.29 versus -2.33±7.67, p=0.000).

**Table 6:** Comparison of fall in ∆ SBP during lying to standing test in chronic coronary syndrome patients pre- and post-PCI The values are expressed as mean ± SD. *a p-value<0.05 was considered statistically significant. ∆ SBP: delta systolic blood pressure; PCI: percutaneous coronary intervention

Parameter	pre-PCI (n=30)	post-PCI (n=30)	p-value
∆ SBP (mmHg)	-20.73± 10.29	-2.33± 7.67	0.000*

## Discussion

The parasympathetic reactivity has been tested by comparing the effect of deep breathing on the R-R interval (E:I ratio), the effect of Valsalva manoeuvre on the R-R interval (Valsalva ratio) and the effect of sudden standing on the R-R interval (30:15 ratio). The E:I ratio and delta HR in the deep breathing test, as well as the Valsalva ratio and 30:15 ratio during the lying to standing test, were significantly lower in CCS patients pre-PCI compared to post-PCI, showing a reduced parasympathetic reactivity in CCS patients. To our knowledge, no previous study has looked at cardiovascular reactivity in CCS patients and how revascularization affects it. Our findings of decreased parasympathetic response to various stress tests in CCS patients prior to coronary revascularization are consistent with lower parasympathetic tone as shown by lower HF and RMSSD in patients prior to PCI [10].

Immediate standing causes a rise in heart rate, which peaks between the 10th and 15th beat. Following that, the HR drops to a minimum in 1 to 2.5 minutes, then increases again in 2.5 to 4 minutes to stabilise (the steady state heart rate) [12]. Prior to coronary revascularization, the maximum increase in heart rate in CCS participants was much lower, according to our findings. The rise in heart rate that occurs when a person rises from a supine position is mediated by the baroreceptor reflex, which evaluates the autonomic nervous system's integrity [13]. Similarly, the integrity of the baroreceptor reflex arc is tested by the heart rate response to the Valsalva manoeuvre. Changes in vagal efferent innervation to the heart, as well as sympathetic vasomotor activity, occur during and after the Valsalva manoeuvre, owing to stimulation of both carotid and aortic baroreceptors, as well as other intrathoracic stretch receptors [12].

In CCS patients, a substantial increase in the E:I ratio and delta HR was found in the deep breathing test after PCI. The sympathetic supply to the heart diminishes during inspiration, whereas the parasympathetic supply increases [13]. The heart rate elevates during inspiration as a result of this change in innervations to the heart. Expiration triggers the opposite mechanism, resulting in a reduction in heart rate. Sinus arrhythmia is defined as a change in heart rate that happens during distinct phases of respiration, particularly the increase in heart rate during deep inhalation [13]. This arrhythmia phenomenon is intensified by deep breathing. Increased parasympathetic reactivity indicates an improvement in the E:I ratio and delta HR after PCI.

Thus, our findings support the discovery of diminished vagal regulation of heart rate in CCS patients and its subsequent restoration by PCI revascularization in these subjects [10].

The sympathetic reactivity was assessed by contrasting the change in diastolic blood pressure during an isometric handgrip test with the change in systolic blood pressure during a lying to standing test. CCS patients had lower systolic blood pressure on standing and a lower rise in diastolic blood pressure in response to continuous isometric contraction before coronary revascularization, indicating a reduced sympathetic reactivity to stress tests. Furthermore, we discovered a significant improvement in sympathetic reactivity in CCS patients following revascularization, which is consistent with the findings of higher LF in the post-PCI patients of CCS [10]. The autonomic tone and flexibility of the blood arteries play a role in the drop in SBP from lying to standing. When a person stands up, venous pooling occurs, causing a drop in blood pressure. This drop is reversed by baroreceptor activation and a reflex increase in sympathetic activity, which causes blood vessels to constrict [13].

Abdelnaby et al. found that dysfunction of the segmental section of the left ventricle contributed to the imbalance of the sympatho-vagal system in CCS patients, and that intervention with balloon angioplasty (PTCA) reduced HRV and left ventricle dysfunction [14]. These data support the theory that when cardiac function is disrupted, afferent sympathetic mechanoreceptors are discharged, resulting in heart rate autonomic control impairment. They also discovered that following PTCA, autonomic functions were improved. Our research yielded comparable results.

Aside from left ventricle dysfunction, stress indices of the systolic and diastolic wall defined the imbalance between the sympatho-vagal system synergistically in patients with single vessel CCS, and when the left ventricle dysfunction was rectified, indices of wall stress improved HRV. Changes in cardiac function and ventricular wall stress mostly cause efferent vagal and sensory sympathetic afferent fibres to discharge, modulating long-term rather than short-term heart rate changes [9].

Aydinlar et al. [15] observed that myocardial ischaemia is caused by the activation of mechanoreceptors and chemoreceptors in the ventricular wall and that the stimulation of these ventricular receptors is mostly caused by the blockage of coronary arteries [15]. Changes in coronary blood flow, as well as mechanical stretch, are reported to stimulate the mechanoreceptors in the left coronary artery, which can lead to a reflexive decrease in sympathetic drive [15]. In contrast, blockage of the left coronary artery stimulates mechanoreceptors and chemoreceptors in the ventricular wall, which enhances activity in sympathetic efferent axons that travel toward the heart in an experimental setting [15].

Regarding this study's limitations, we would advise extensive longitudinal research with a larger sample size to get more definitive findings. The study's patient population was small. It is crucial to do a study that exclusively includes women because the study population primarily consisted of men with little representation of women. More research should be done in this area because there have been conflicting findings regarding autonomic reactivity in CCS patients pre PCI and post PCI and very few studies have been conducted in the Indian community.

To summarise, our findings show that CCS patients have an autonomic deficit before PCI coronary revascularization, with a significant loss of parasympathetic and sympathetic tone. Furthermore, autonomic balance was restored following revascularization, implying autonomic modulation in CCS patients.

## Conclusions

Based on our findings, we conclude that autonomic dysfunction existed in chronic coronary syndrome prior to percutaneous coronary intervention and that there was significant cardiovascular autonomic modulation following revascularization procedures such as percutaneous coronary intervention, indicating a restoration of both basal cardiac autonomic tone and autonomic reactivity. One of the most efficient and gold-standard methods for measuring autonomic dysfunction is to use cardiovascular reflex tests. More studies are needed to clarify the effectiveness of these tests in clinical care, as well as their utility in early identification and as an independent prognostic factor for CCS.
